# Context-Aware Integrated Navigation System Based on Deep Learning for Seamless Localization

**DOI:** 10.3390/s24237678

**Published:** 2024-11-30

**Authors:** Byungsun Hwang, Seongwoo Lee, Kyounghun Kim, Soohyun Kim, Joonho Seon, Jinwook Kim, Jeongho Kim, Youngghyu Sun, Jinyoung Kim

**Affiliations:** Department of Electronic Convergence Engineering, Kwangwoon University, Seoul 01897, Republic of Korea; hbsun1225@kw.ac.kr (B.H.); swoo1467@kw.ac.kr (S.L.); sdrudgnsdl@kw.ac.kr (K.K.); kimsoogus@kw.ac.kr (S.K.); dimlight13@kw.ac.kr (J.S.); yoonlight12@kw.ac.kr (J.K.);jh980828@kw.ac.kr (J.K.); yakrkr@kw.ac.kr (Y.S.)

**Keywords:** seamless localization, context-aware, integrated navigation system, kalman filter, deep learning

## Abstract

An integrated navigation system is a promising solution to improve positioning performance by complementing estimated positioning in each sensor, such as a global positioning system (GPS), an inertial measurement unit (IMU), and an odometer sensor. However, under GPS-disabled environments, such as urban canyons or tunnels where the GPS signals are difficult to receive, the positioning performance of the integrated navigation system decreases. Therefore, deep learning-based integrated navigation systems have been proposed to ensure seamless localization under various positioning conditions. Nevertheless, the conventional deep learning-based systems are applied with a lack of consideration of context features on surface condition, wheel slip, and movement pattern, which are factors causing positioning performance. In this paper, a context-aware integrated navigation system (CAINS) is proposed to ensure seamless localization, especially under GPS-disabled conditions. In the proposed CAINS, two deep learning layers are designed with context-aware and state estimation layers. The context-aware layer extracts vehicle context features from IMU data, while the state estimation layer predicts the GPS position increments by modeling the relationship between context features, velocity, attitude, and position increments. From simulation results, it is confirmed that the positioning accuracy can be significantly improved based on the proposed CAINS when compared with conventional navigation systems.

## 1. Introduction

Localization has been recognized as a key technology of information and communication technology (ICT) for providing seamless location-based service (LBS) [1,2,3]. Global positioning systems (GPSs), inertial measurement units (IMUs) and odometer sensors are widely used for various applications such as autonomous navigation, search and rescue (SAR) and target monitoring [4]. The IMU provides continuous estimates of navigation information from inertial data such as acceleration, angular velocity, and magnetic field strength. However, the inherent bias and random walk noise in measurements from IMU can cumulatively increase the detection error of navigation information [5]. The odometer sensor measures the vehicle’s traveled distance and velocity to estimate positions, complementing the inertial data by tracking wheel rotations. However, the odometer sensor is susceptible to cumulative errors due to wheel slip or uneven terrain. Therefore, integrated navigation systems that combine different sensors from different sources have been used to complement the estimated positioning of each sensor.

A Kalman filter (KF) has been widely used in integrated navigation systems. The linear KF is a linear quadratic estimation filter based on Gaussian noise, estimating the current states of a system over time by recursively processing input measurements within a mathematical model. As the linear KF estimates the hidden state from noisy measurements in real time, it is a key component in integrating different navigation systems [6,7]. The difficulty in adapting the linear KF to non-linear systems has led to the proposal of alternative filters, namely the extended Kalman filter (EKF) and the unscented Kalman filter (UKF), which are suited to addressing non-linear systems [8,9]. However, these filters, which are model-based algorithms dependent on prior knowledge and mathematical models, often encounter performance degradation when it is difficult to define an accurate mathematical model or when prior information is partial. The GPS information may be partially received or unavailable under GPS-disabled conditions where satellite signals cannot be received, such as in urban canyons and tunnels [10,11]. Under GPS-disabled conditions, navigational information such as position and velocity may be unavailable, and it may be complicated to establish an accurate mathematical model. Various solutions without deep learning approaches have been proposed for path estimation under these conditions. However, a deep learning-based approach has been selected as a promising solution. This is because the deep learning-based approaches are capable of modeling and adapting to non-linear relationships between sensor measurement relationships that traditional model-based methods have been unable to capture effectively. Deep learning offers the flexibility to learn these patterns directly from sensor data, decreasing the need for precise mathematical models that may not fully capture real-world nonlinearities. By incorporating deep learning with the Kalman filter, this approach can dynamically predict input states and adjust the filter parameters based on learned sensor characteristics, thereby enhancing the accuracy and robustness of the integrated navigation system under challenging conditions [12,13].

To enhance accuracy and robustness in non-linear models, combining the KF with the deep learning approaches has been proposed [14,15,16,17,18,19,20,21]. In [14], a multi-layer perceptron (MLP)-based state estimation method has been utilized to handle GPS-disabled conditions. However, MLP-based methods often struggle to extract complex and dynamic features. In addition, their estimation error may increase as the GPS is disabled for prolonged periods. A fuzzy neural network-based state estimation model [15] was proposed to mitigate the increasing errors in longer GPS-disabled states, and an ensemble learning algorithm [16] has been proposed to extract complex features from IMU sensors. In [17], IONet has been introduced, a sequential learning framework designed to reduce drift in inertial odometry using RNNs. However, this approach primarily focuses on trajectory prediction from raw IMU data, limiting its adaptability to diverse real-world conditions. Conventional approaches predict the position without considering vehicle context information. The vehicle context information includes surface conditions, movement patterns, and wheel slip, leading to increased navigation errors under certain circumstances. The proposed navigation system overcomes these limitations by integrating context-aware features, ensuring robust navigation performance in complex scenarios.

A recurrent neural network (RNN)-based state estimation model has been proposed to deal with long-term GPS-disabled conditions [18]. This approach can seamlessly provide accurate navigation information using RNN, which can learn the intricate patterns and correlations between sensor measurements over time. RNNs are mainly used to analyze sensor data with temporal features [22] by leveraging temporal dependencies through a hidden layer that extracts features from previous inputs. However, as the length of the sequence increases, RNNs encounter gradient vanishing issues. Long short-term memory (LSTM) algorithms have been proposed to address this issue [23]. Despite these advancements, traditional RNN- and LSTM-based state estimation models are limited in addressing the diverse sensor characteristics inherent in navigation systems, as spatial dependencies between sensor data are not considered. The state estimation model incorporating a convolutional neural network (CNN) and gated recurrent unit (GRU) was introduced in [19]. CNN can effectively extract spatial features across different sensors and analyze potential error information from sensor data. Furthermore, the GRU, which employs fewer parameters than the LSTM, can reduce the computational load for the seamless integrated navigation system. In [20], RoNIN has been proposed, leveraging temporal convolutional networks (TCNs) and LSTMs to improve inertial navigation accuracy under diverse device placements. In [21], a graph-based state estimation model is proposed to design a robust state estimation model in perceptually degraded environments. However, [20,21] primarily focuses on inertial data without explicitly incorporating spatial features or adapting to multi-sensor navigation tasks, limiting its flexibility in more complex real-world scenarios.

In this paper, a novel scheme called “context-aware integrated navigation system (CAINS)” is proposed to address the limitations caused by the lack of context-awareness, which affects GPS positioning accuracy. The context-awareness refers to the ability to recognize environmental information that can potentially affect an increase in navigation errors, such as surface conditions, movement patterns, and wheel slip. Through the incorporation of environmental information, the estimated positioning in the CAINS can be proactively adjusted by context awareness. The proposed CAINS uses GPS, IMU, and odometer sensors to model the integrated navigation system. Furthermore, the proposed CAINS deep learning model, consisting of context-aware and state estimation layers, is employed to achieve seamless localization under GPS-disabled conditions. The context-aware layer extracts the vehicle context features from IMU data. The state estimation layer predicts the GPS position increment by modeling the relation among the context features, velocity, attitude, and position increment. The predicted GPS position is employed as measurement data for the KF to complement the estimated position in the IMU and odometer sensors. The main contributions of this paper are summarized as follows:

- The proposed CAINS is proposed to reduce estimation error, especially under GPS-disabled conditions. In the proposed CAINS, two distinct deep learning layers are incorporated with context-aware and state estimation layers. In context-aware layers, the features related to a vehicle state are extracted by reflecting the changes in sensor measurements within the changeable environment. Additionally, vehicle context features and navigation information are used to predict the GPS position information in the state estimation layer. Therefore, the context-aware layer can effectively complement the state estimation layer, which can achieve seamless localization with context-awareness.
- The proposed CAINS is designed to ensure seamless localization, operating effectively in switching between GPS-enabled and GPS-disabled conditions. Additionally, to account for practical navigation constraints, each sensor should operate at a different sampling rate and under certain conditions, which makes it unable to take measurements. To consider these operating constraints, the proposed CAINS is designed to enable sensor fusion even in situations where sampling rates are different and sensors are not operational, thereby achieving seamless localization.
- Validation of positioning performance is required to use data collected from diverse environments, including movement patterns, various surfaces, GPS-enabled and GPS-disabled conditions. The University of Michigan North Campus Long-Term (NCLT) dataset has been well known for encompassing both indoor and outdoor environments, including streets, sidewalks, and passageways. Therefore, the NCLT dataset is used in this paper to evaluate various positioning conditions. Using realistic datasets, the proposed CAINS demonstrates its ability to achieve seamless localization across diverse real-world environments.

The remainder of this paper is organized as follows: Section 2 provides the system model to help understand the proposed integrated navigation system. In Section 3, the proposed CAINS is described in detail. Simulation results are shown in Section 4. Finally, Section 5 presents the conclusions of this paper.

## 2. System Model

**Figure 1:**
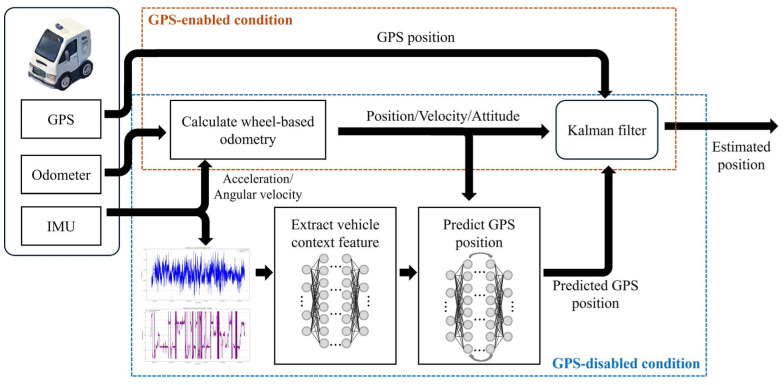
Schematics for the proposed CAINS.

The proposed CAINS is designed to provide seamless localization by fusing GPS, IMU and odometers, while concurrently leveraging deep learning techniques to enhance navigation accuracy under GPS-disabled conditions. The proposed CAINS is adapted to varying environments through a context-aware mechanism that accounts for environmental factors and a state estimation layer by which position increments are predicted based on sensor data and context features. The vehicle’s position is estimated by processing continuous IMU and odometer data, with errors corrected by GPS when available. Under GPS-disabled conditions, the deep learning model predicts the GPS position using historical IMU and odometer data. The schematics for the proposed CAINS are shown in Figure 1. In the proposed CAINS, IMU and odometer data are utilized to provide precise estimated positions, even under GPS-disabled conditions.

### 2.1. Kalman Filter

**Figure 2:**
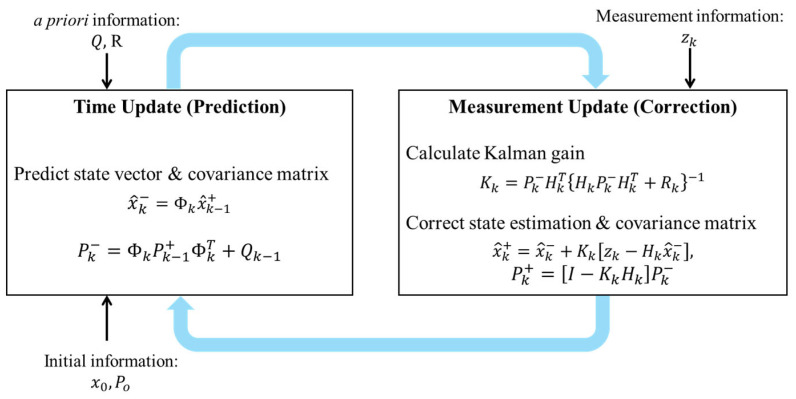
Time and measurement update phases in the Kalman filter.

The KF operates as a recursive filtering algorithm, estimating the system state by integrating measurements from various sensors. In the navigation system, state information primarily consists of physical quantities such as position, velocity, acceleration, and angular velocity. The KF estimates the current state through two main phases: the time update and the measurement update phases. These update phases are shown in Figure 2. During the time update phase, the current state, including position, velocity, and heading angle, along with the associated covariance are predicted as the following equation:

(1) $${\hat{x}}_{k}^{-}=\Phi_{k}{\hat{x}}_{k-1}^{+},$$

(2) $$P_{k}^{-}=\Phi_{k}P_{k-1}^{+}\Phi_{k}^{T}+Q_{k-1},$$

where predicted and corrected vectors are represented by the superscript ‘−’ and ‘+’, respectively. ${\hat{x}}_{k}$ is the state vector at time k, representing the estimated state of the vehicle. $\Phi_{k}$ is the state transient matrix that represents the given mathematical model. $Q_{k}$ is the system noise matrix, modeling unexpected variations in the vehicle’s motion or sensor performance in the filtering process. $P_{k}$ is a covariance matrix representing the uncertainty in state estimation. In the measurement update phase, the measurement vector is used to update the predicted state vector and covariance matrix, as follows:

(3) $$K_{k}=P_{k}^{-}H_{k}^{T}{\left\{H_{k}P_{k}^{-}H_{k}^{T}+R_{k}\right\}}^{-1},$$

(4) $${\hat{x}}_{k}^{+}={\hat{x}}_{k}^{-}+K_{k}\left[z_{k}-H_{k}{\hat{x}}_{k}^{-}\right],$$

(5) $$P_{k}^{+}=\left[I-K_{k}H_{k}\right]P_{k}^{-},$$

where $K_{k}$ is Kalman gain, $z_{k}$ is the measurement vector containing the sensor measurements used to update and correct this state estimate, $H_{k}$ is the observation matrix, mapping the predicted state variables to the measurement space allowing comparison with the measurement vector, $R_{k}$ is the measurement noise matrix, representing the errors and uncertainties inherent in sensor measurements during the filtering process, I is the identity matrix. The Kalman gain is the weight that adjusts between estimated state and measurement vectors based on a priori covariance *P* and measurement noise covariance R. When the measurement vector $z_{k}$ is obtained, it is compared with the predicted measurement vector $H\hat{x}$. The definition and role of variables in the KF are summarized in Table 1.

### 2.2. Mathematical Model for Integrated Navigation System

The odometer and IMU sensor are used to design a wheel-based odometry system. To integrate various sensors operating in different frames, each body frame is transformed into a global frame. The body frame refers to the coordinate system that is fixed to the vehicle as it moves and rotates, while the global frame is an external, fixed coordinate system that represents the entire environment. The state vector of the global frame is expressed as follows:

(6) $$x_{k}=[P_{north},\;P_{east},\;v_{north},\;v_{east},\;\theta ],$$

where $P_{north}$ and $P_{east}$ are position of north and east direction, respectively. $v_{north}$ and $v_{east}$ are velocity of each direction. θ represents heading angle which is measured from IMU sensor. As the wheel velocities are obtained from odometer, center velocity can be calculated from the following equation.

(7) $$v_{c}=\frac{v_{r}+v_{l}}{2},$$

where $v_{r}$ and $v_{l}$ are represented velocity of right wheel and left wheel, respectively. From the center velocity $v_{c}$ and heading angle θ, velocity of global frame can be expressed as follows:

(8) $$v_{north}=v_{c}cos\theta ,\;v_{east}={-v}_{c}sin\theta .$$

**Figure 3:**
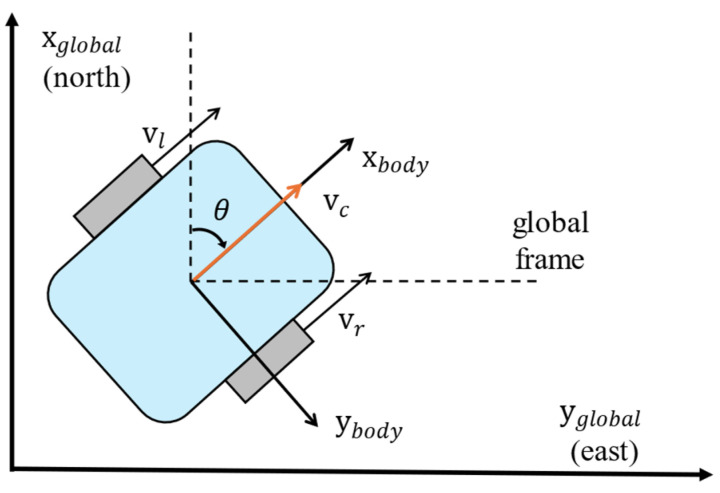
Coordinate for vehicles in global frames.

The robot coordinates in the global frame are depicted in Figure 3. The wheel-based odometry is given as,

(9) $${\hat{x}}_{k}^{-}=\Phi_{k}x_{k-1}^{+}=\left[\begin{array}{c} \begin{array}{c} \begin{array}{c} P_{north}+v_{north}∆t\\ P_{east}+v_{east}∆t\\ v_{north} \end{array}\\ v_{east} \end{array}\\ \theta_{k} \end{array}\right],$$

where ∆t is sampling time. The GPS sensor provides the measurement vector. The measurement vector and observation matrix are described as follows:

(10) $$z_{k}=\left[\begin{array}{c} P_{north}^{GPS}\\ P_{east}^{GPS} \end{array}\right],$$

(11) $$H=\left[I_{2\times 2},\;O_{2\times 3}\right],$$

where $P_{north}^{GPS}$ and $P_{east}^{GPS}$ are measured position from the GPS sensor. Matrix I and O are identity and zero matrices, respectively.

## 3. Context-Aware Integrated Navigation System

**Figure 4:**
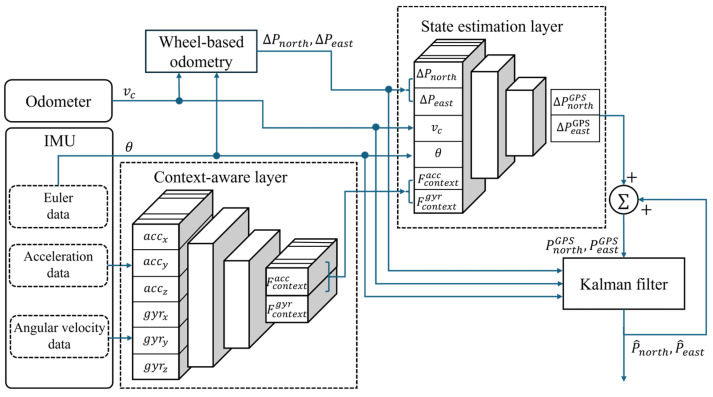
Structure of the proposed CAINS.

The proposed CAINS aims to implement an integrated navigation system that operates effectively in both GPS-enabled and GPS-disabled conditions. The deep learning model of the proposed CAINS consists of a context-aware layer and a state estimation layer. The context-aware layer extracts the vehicle context feature by learning the vehicle’s vibration and movement state information through acceleration and angular velocity data. The state estimation layer predicts the position information of the GPS through the extracted context information and navigation information of the vehicle. The state estimation layer is trained when the GPS is enabled to fuse with the odometer and IMU sensor. With the state estimation layer, the proposed CAINS predicts the GPS position by defining a non-linear mathematical model based on the deep learning algorithm. In order to define the non-linear mathematical model, it is necessary to select key factors that can explain the GPS position. According to [24], the increment of position from the GPS can be described as follows:

(12) $$∆P^{GPS}=\int \int (C_{b}^{n}f_{ib}^{b}\left(t\right)-(2\omega_{ie}^{n}\left(t\right)+\omega_{en}^{n}\left(t\right))\times V_{n}\left(t\right)+G_{n})dtdt,$$

where C is the direction cosine matrix, f is the specific force measured from the accelerometer, $\omega_{ie}$ and $\omega_{en}$ are represented angular rate of earth frame *e* to the inertial frame and the angular rate of the navigation frame *n* to the earth frame *e*, V  is the velocity of the vehicle, G is the gravity vector. As the $G_{n}$ is affected by the longitude and latitude, which lead to small changes, it is not utilized as input data. Position-increment/velocity/heading/context-feature data are trained to estimate the position increment from the GPS. The input and output vector of the state estimator are described as

(13) $$Input:\;[∆P_{north}^{odometry},\;∆P_{east}^{odometery},\;v_{c},\;\theta ,\;F_{context}^{acc},\;F_{context}^{gyr}],$$

(14) $$Output:\left[∆P_{north}^{GPS},\;∆P_{east}^{GPS}\;\right],$$

where $F_{context}^{acc}$ and $F_{context}^{gyr}$ represent context features from acceleration and angular velocity extracted from the context-aware layer. Figure 4 shows input and output structures for the deep learning model in the proposed navigation system. Under GPS-disabled conditions, an increment of position $∆P^{GPS}$ can be estimated only by IMU and odometer. The estimated GPS position can be calculated as follows:

(15) $$P^{GPS}=P^{GPS_{0}}+\sum_{i=0}^{t}∆P^{GPS_{i}},$$

where $P^{GPS_{0}}$ is the last position just before the GPS signal was lost and, $∆P^{GPS_{i}}$ is the increment of the position predicted by the state estimation layer during the time the GPS signal was lost.

**Figure 5:**
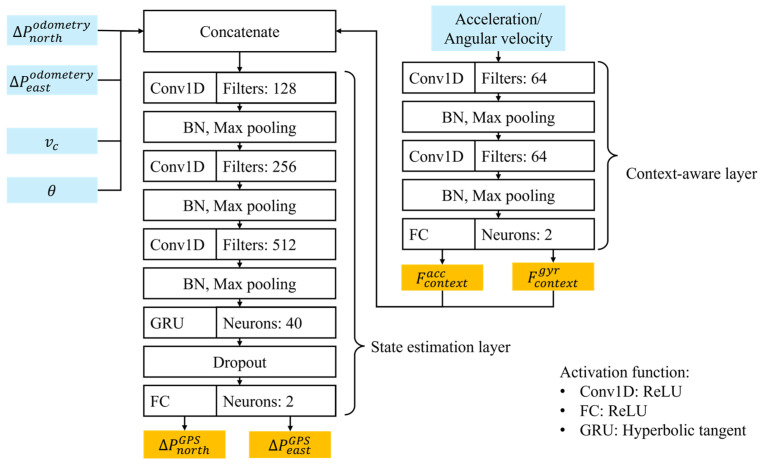
Schematic diagram of context-aware and state estimation layers.

As shown in Figure 5, the context-aware and state estimation layers mainly consist of convolutional 1D (Conv1D), GRU, and fully connected (FC) layers. The hyperparameter settings used for simulation are presented in Table 2. The context-aware layer extracts information on the vehicle states and sensor environmental characteristics using Conv1D, batch normalization (BN), and max pooling layers based on the IMU’s acceleration and angular velocity data. The Conv1D is used to capture relationships between features across different sensor channels by applying convolutional filters that identify spatial dependencies among sensor inputs [25]. The convolutional filters can capture relationships in acceleration and angular velocity, such as abrupt changes in motion or rotational speed, which are crucial for understanding the vehicle’s behavior and its interaction with the environment. In the state estimation layer, spatial features are extracted from navigation data measured by the IMU and odometer sensor using Conv1D, BN, and max pooling layers to capture the spatial dependencies that influence changes in the GPS positioning. Additionally, the GRU layer extracts temporal features from sequential data while addressing the vanishing gradient problem common in long sequences. The GRU is designed to selectively update and reset information as needed, which helps it retain essential temporal features over long sequences and prevents the loss of temporal features due to vanishing gradient problems. This design uses fewer parameters than LSTM, making it more computationally efficient while preserving accuracy [26]. Finally, the updated GPS information is output through the fully connected (FC) layer. Algorithm 1 shows the practical implementation for Kalman filtering.
**Algorithm 1** Practical implementation for Kalman filteringLoad odometer, GPS, IMU data$\mathrm{Initialize}\;\mathrm{system}\;\mathrm{parameters}\;\mathrm{and}\;\mathrm{matrixes},\;\mathrm{initial}\;\mathrm{state}\;\mathrm{vector}\;x_{0}$, covariance matrix P, noise matrixes Q and R, transient matrix Φ, observation matrix H**Kalman filtering:****for** each time step **do** $\mathrm{Compute}\;\mathrm{wheel}\;\mathrm{velocity}\;v_{C}$ by Equation (7) Calculate the vehicle’s position incrementally by Equation (8) Find GPS and IMU data with the closest timestamp to the odometer **Time update phase:**  Update the state vector and covariance matrix  Create time series data for inputting deep learning model **Measurement update phase:**  **if** GPS is disabled for longer than threshold (0.3 s) **then**   Input time series data to the pre-trained model to predict the change in    $\mathrm{GPS}\;\mathrm{position}\;\mathrm{information}\;∆P_{north}$$\;\mathrm{and}\;∆P_{east}$  Update Kalman gain K  Calibrate the state vector by Equation (9) **Return** calibrated navigation information

## 4. Simulation Results

### 4.1. Simulation Setting

The University of Michigan NCLT dataset is utilized to train and evaluate the proposed navigation system [27]. The NCLT dataset is composed of 27 sessions measured over 15 months, capturing a wide range of environments and conditions. The Segway robotic platform provides navigation information from multi-sensors, such as a real-time kinematic (RTK), a consumer-grade GPS, an omnidirectional camera, 2D/3D light-detection-and-ranging (LIDAR), IMU, a single-axis fiber optic gyroscope (FOG), and odometer. In the NCLT dataset, various environmental conditions are included such as different terrains, streets, sidewalks, pathways, and diverse weather conditions. Therefore, the generalizability and adaptability of the proposed navigation system can be evaluated across various scenarios. The sampling rates of the GPS, IMU, and odometer sensors are 5 Hz, 37 Hz, and 47 Hz, respectively. For GRU-based models, the input window size is an important factor when extracting temporal features. Considering the sampling rate of the IMU sensor, the window size was set to 7. Table 3 shows the specifications of navigation sensors used in the simulation: the GPS and IMU data were obtained from a Microstrain 3DM-GX3-45 IMU sensor, while odometer data were provided by a Segway wheel encoder [27].

### 4.2. Performance Evaluation

The performance of the proposed navigation system was compared with three different navigation systems: (1) wheel-based odometry; (2) only the state estimation layer with integrated navigation system (OSEINS); (3) the proposed CAINS. A comparison was performed to assess the effectiveness of the context-aware layer between a model using only the state estimation layer and one incorporating both the context-aware and state estimation layers. The maximum, mean, standard deviation errors and root mean square error (RMSE) are calculated to evaluate the performance of each navigation system. Additionally, 95% confidence intervals (CIs) for the mean error were computed to assess the statistical significance of the results.

**Figure 6:**
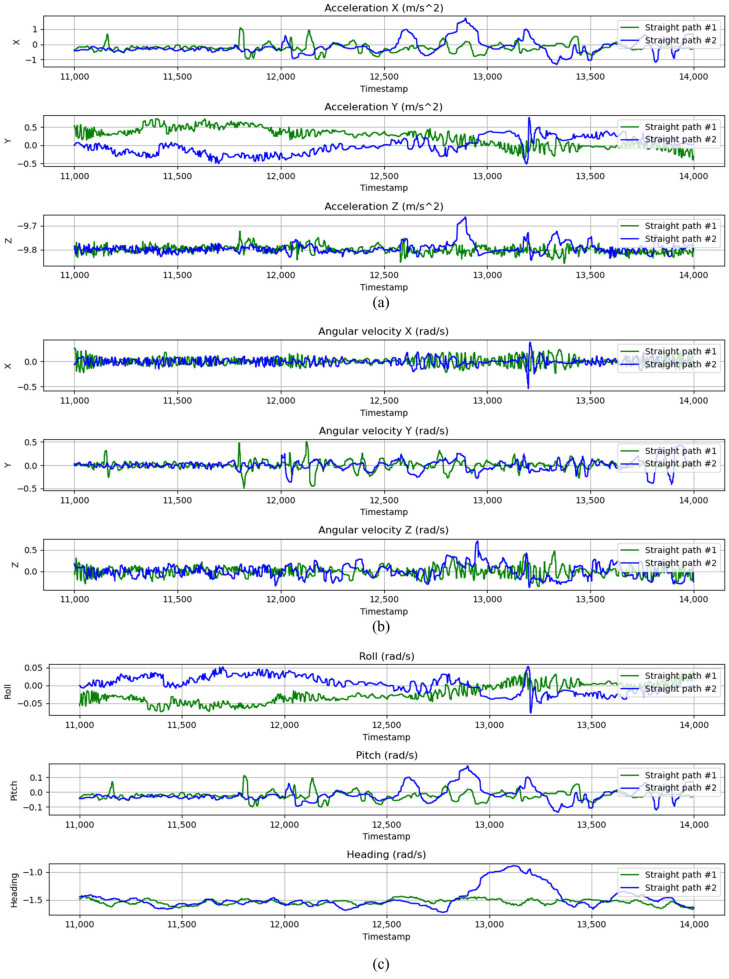
Comparison of IMU data in straight paths; (**a**) acceleration data, (**b**) angular velocity data, and (**c**) attitude data.

In order to verify the performance of the context-aware layer, IMU data for two different paths was analyzed. A comparison of the IMU data for straight-line motion on both paths is shown in Figure 6. Since both paths are followed in the same direction, similar patterns are shown in the angular velocity graphs. However, between timestamps 12,500 and 13,000, violent fluctuations in the acceleration data indicate that an impact on the vehicle was experienced. This impact may have been caused by perturbations in the vehicle’s motion, as seen in the angular velocity and attitude data, particularly in the heading, which shows a significant deviation during this period. The violent fluctuation in heading indicates a disturbance that could affect the accuracy of the navigation system, potentially leading to positioning errors.

**Figure 7:**
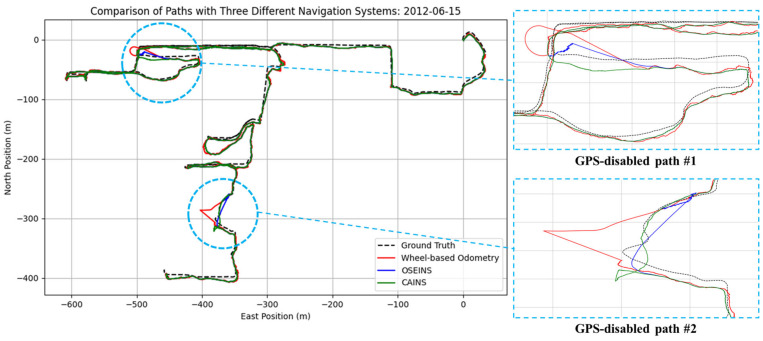
Comparison of paths with three different navigation systems.

**Figure 8:**
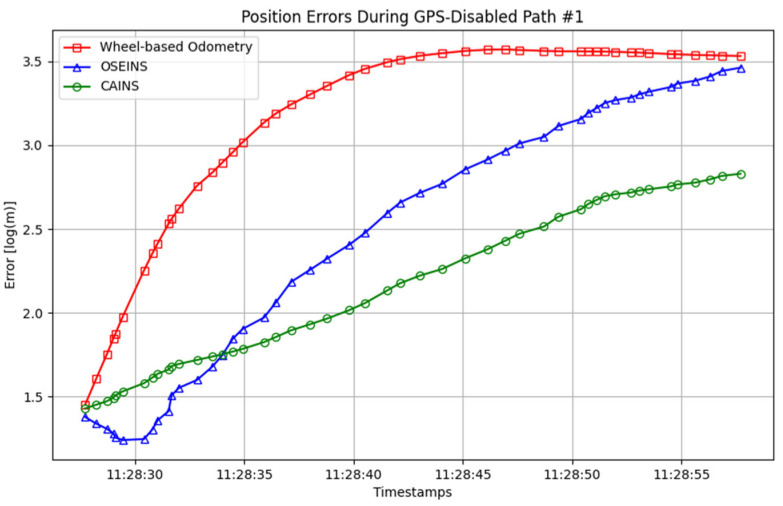
Position errors during GPS-disabled path #1.

**Figure 9:**
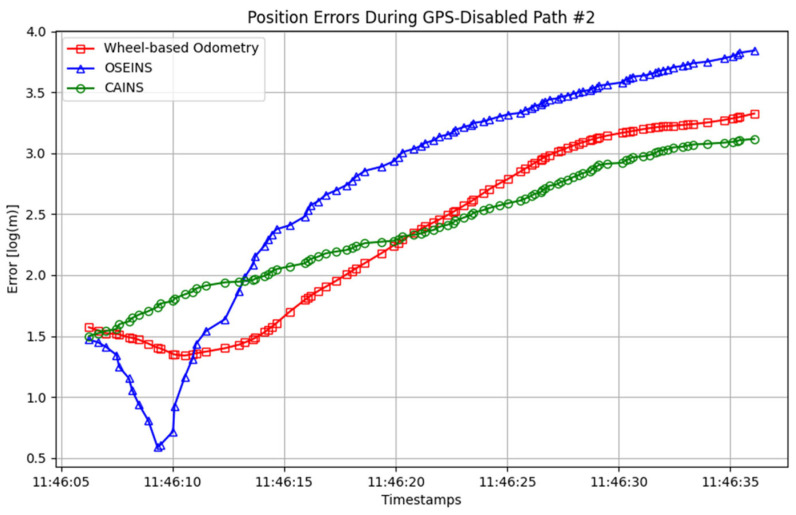
Position errors during GPS-disabled path #2.

**Figure 10:**
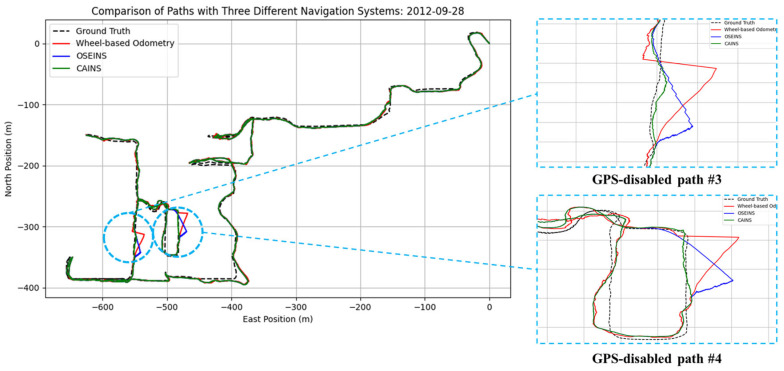
Comparison of paths with three different navigation systems.

**Figure 11:**
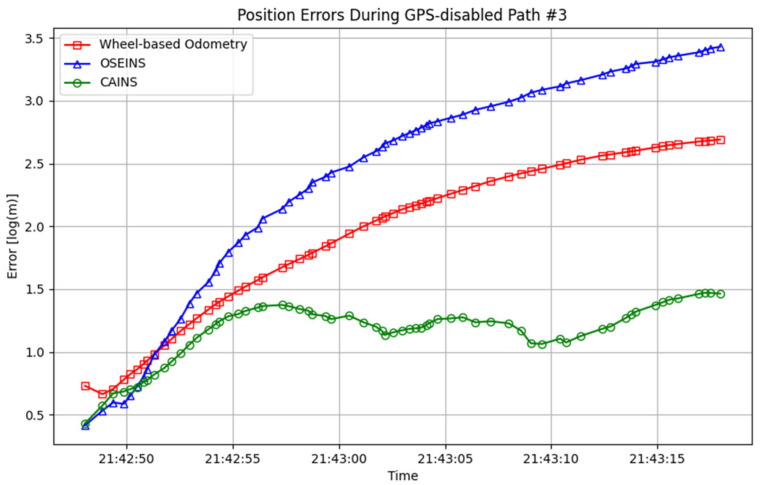
Position errors during GPS-disabled path #3.

**Figure 12:**
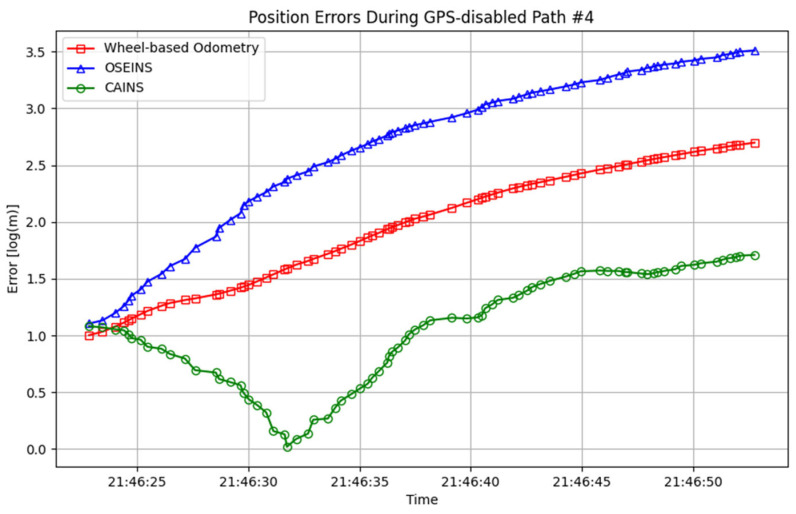
Positioning errors during GPS-disabled path #4.

A comparison of paths with three different navigation systems is presented in Figure 7, Figure 8, Figure 9, Figure 10, Figure 11 and Figure 12, with data collected on the dates 15 June 2012 and 28 September 2012, respectively. For each date, entire paths are showed in Figure 7 and Figure 10, two GPS-disabled paths were evaluated, and the errors in these paths are shown in Figure 8, Figure 9, Figure 11 and Figure 12. The errors in each GPS-disabled path are summarized in Table 4, Table 5, Table 6 and Table 7. When examining Figure 8 and Figure 9, it has been confirmed that the positioning errors of all three navigation systems increase continuously over time. However, the error of the proposed CAINS was observed to increase in a relatively smooth curve. Despite rapid increases in positioning error during GPS-disabled paths, the CAINS and OSEINS have lower error levels, likely due to the state estimation layer’s ability to effectively estimate the increment of the GPS position. Figure 11 and Figure 12 show that the CAINS maintains a lower error level compared with the OSEINS and wheel-based odometry. In particular, the OSEINS can be seen to have the highest position error, suggesting that the vehicle context features may significantly influence the prediction of the GPS position increments. As shown in the results in Table 4, Table 5, Table 6 and Table 7, the RMSE of the CAINS in GPS-disabled path is reduced by up to 68.17% and 86.53%, respectively, compared to wheel-based odometry and OSEINS. The enhanced performance of the proposed CAINS, attributable to its context-aware layer, enables more reliable navigation in dynamic environments, which can be expected to provide a flexible and comprehensive solution for seamless localization.

## 5. Conclusions

In this paper, the CAINS have been proposed to ensure seamless localization for operating effectively in switching under GPS-disabled conditions. The deep learning model in the CAINS consists of two different layers: the context-aware and the state estimation layers. The context-aware layer is mainly composed of the Conv1D layer to extract vehicle context features between spatial dependencies of inertial data, while the state estimation layer is mainly composed of Conv1D and GRU to estimate increments in the GPS position by extracting spatial and temporal dependencies. In order to evaluate the proposed navigation system, the NCLT dataset was utilized which includes various environmental conditions such as different terrains, streets, sidewalks, pathways, and diverse weather conditions. The navigation performances were evaluated in four different GPS-disabled paths. From the simulation results, it was confirmed that the proposed CAINS can improve navigation performance over traditional integrated navigation systems. Furthermore, the ability of CAINS to adapt to diverse terrains and weather conditions demonstrates its potential applicability in real-world scenarios. However, the current CAINS model relies solely on GPS, IMU, and odometer sensors, which may limit its effectiveness in scenarios where these sensors are insufficient or unreliable. Additionally, the incorporation of deep learning models increases the computational load, which could challenge real-time deployment. To address these limitations, future research should focus on integrating auxiliary sensors, such as LiDAR and cameras, to enhance robustness and adaptability across various environments. Optimization techniques for reducing computational complexity and improving real-time performance are also critical for practical applications. Real-time testing on diverse autonomous platforms is necessary to apply to real-world environments, such as rapid changes in vehicle dynamics or unexpected environmental conditions.

For autonomous vehicles, CAINS’s ability to operate accurately under GPS-disabled conditions, such as urban canyons, tunnels, or densely forested areas, has the potential to improve the reliability and safety of navigation systems. In search and rescue operations, CAINS may provide dependable localization in remote or obstructed areas where GPS is unavailable, enhancing mission success rates and responder safety. Beyond these applications, CAINS can be adapted for use across various other domains that require robust navigation in GPS-limited settings.

## Figures and Tables

**Table 1:** Definition of variables in the Kalman filter.

Variable	Definition
x	State vector, containing the current estimated state of the navigation system, such as position, velocity, and orientation, and continuously updated as new data is processed.
P	Covariance matrix, representing the level of uncertainty in the state estimate, which updates over time to reflect the accuracy of the predictions and corrections.
Φ	State transition matrix, representing the mathematical model used to predict the current state of the navigation system (such as position, velocity, and orientation) from the previous state.
H	Observation matrix, mapping the predicted state to the actual measurement space for comparison with measured sensor data.
z	Measurement vector, containing the observed data from sensors, such as GPS and IMU readings, used to correct the predicted state during the measurement update phase.
K	Kalman gain, determining the optimal balance between the predicted state and new measurements during the update step.
R	Measurement noise matrix, representing the errors and uncertainties in sensor measurements.
Q	System noise matrix, modeling unexpected variations in the vehicle’s motion or sensor behavior during the filtering process.

**Table 2:** Hyperparameter settings for CAINS.

Parameter	Value
Batch size	64
Learning rate	0.0001
Training epoch	100
Optimizer	Adam
Loss function	Mean square error

**Table 3:** Specifications of navigation sensors.

Sensor	Specification	Value
IMU	Sampling rate	47 Hz
	Acceleration initial bias error	±0.002 g
	Acceleration noise density	80 μg/√Hz
	Gyroscope initial bias error	±0.25°/s
	Gyroscope noise density	0.03°/s/√Hz
GPS	Sampling rate	5 Hz
	Horizontal position accuracy	<2.5 m
Odometer	Sampling rate	37 Hz

**Table 4:** Statistics errors of different navigation system under GPS-disabled path #1.

Metric	GPS-Disabled Path #1		
	Wheel-Based Odometry	OSEINS	CAINS
Max (m)	34.5693	30.9286	15.9624
Mean (m)	24.5480 ± 0.7025	13.6653 ± 0.0.6202	8.4880 ± 0.2769
Standard deviation (σ)	10.9753	9.6889	4.3262
RMSE (m)	26.8898 ± 0.5370	16.7515 ± 0.5777	9.5269 ± 0.2710

**Table 5:** Statistics errors of different navigation system under GPS-disabled path #2.

Metric	GPS-Disabled Path #2		
	Wheel-Based Odometry	OSEINS	CAINS
Max (m)	27.1189	45.6790	21.5870
Mean (m)	12.8558 ± 0.3761	21.6461 ± 0.6141	11.5314 ± 0.2504
Standard deviation (σ)	8.3224	13.5885	5.5403
RMSE (m)	15.3145 ± 0.3372	25.5578 ± 0.5461	12.7933 ± 0.2504

**Table 6:** Statistics errors of different navigation system under GPS-disabled path #3.

Metric	GPS-Disabled Path #3		
	Wheel-Based Odometry	OSEINS	CAINS
Max (m)	13.7765	30.0420	3.3596
Mean (m)	6.9750 ± 0.2239	13.2925 ± 0.4951	2.2965 ± 0.0349
Standard deviation (σ)	4.1285	9.1314	0.6439
RMSE (m)	8.1044 ± 0.2078	16.1248 ± 0.4523	2.3850 ± 0.0320

**Table 7:** Statistics errors of different navigation system under GPS-disabled path #4.

Metric	GPS-Disabled Path #4		
	Wheel-Based Odometry	OSEINS	CAINS
Max (m)	13.8911	32.6795	4.5428
Mean (m)	7.1290 ± 0.1851	16.6634 ± 0.4548	2.1561 ± 0.0686
Standard deviation (σ)	3.7309	9.1663	1.3829
RMSE (m)	8.0458 ± 0.1768	19.0168 ± 0.4172	2.5612 ± 0.0680

## Data Availability

Publicly available datasets were analyzed in this study. This data can be found here: https://robots.engin.umich.edu/nclt/, accessed on 29 November 2024.
